# Up-regulation of human cervical cancer proto-oncogene contributes to hepatitis B virus-induced malignant transformation of hepatocyte by down-regulating E-cadherin

**DOI:** 10.18632/oncotarget.5039

**Published:** 2015-09-04

**Authors:** Junfeng Li, Xiaopeng Dai, Hongfei Zhang, Wei Zhang, Shihui Sun, Tongtong Gao, Zhihua Kou, Hong Yu, Yan Guo, Lanying Du, Shibo Jiang, Jianying Zhang, Yusen Zhou

**Affiliations:** ^1^ State Key Laboratory of Pathogen and Biosecurity, Beijing Institute of Microbiology and Epidemiology, Beijing, China; ^2^ Laboratory of Viral Immunology, Lindsley F. Kimball Research Institute, New York Blood Center, New York, NY, USA; ^3^ Key Laboratory of Medical Molecular Virology of Ministries of Education and Health, Shanghai Medical College, Fudan University, Shanghai, China; ^4^ Department of Biological Sciences, The University of Texas at El Paso, El Paso, TX, USA

**Keywords:** hepatocellular carcinoma, human cervical cancer proto-oncogene, hepatitis B virus, TCF/β-catenin pathway, E-cadherin

## Abstract

Hepatocellular carcinoma (HCC) is one of the most fatal human malignancies, Human cervical cancer proto-oncogene (HCCR) aberrantly expressed in a number of malignant tumors, including HCC. HCC is associated with Hepatitis B virus (HBV) infection in a large percentage of cases. To explore the regulation and function of HCCR expression in the development of HCC, we detected HCCR expression in HBV expressing hepatocytes. Results showed that the expression of HCCR was higher in HBV-expressing hepatocytes than that in control cells. Examining different components of HBV revealed that the HBx promotes HCCR expression in hepatocytes via the T-cell factor (TCF)/β-catenin pathway. HCCR expression in HBx transgenic mice increased with as the mice aged and developed tumors. We also found that overexpression of HCCR in hepatocytes promoted cell proliferation, migration, and invasion and reduced cell adhesion. Suppressing HCCR expression abolished the effect of HBx-induced hepatocyte growth. In addition, HCCR represses the expression of E-cadherin by inhibition its promoter activity, which might correlate with the effects of HCCR in hepatocytes. Taken together, these results demonstrate that HBx-HCCR-E-cadherin regulation pathway might play an important role in HBV-induced hepatocarcinogenesis. They also imply that HCCR is a potential risk marker for HCC and/or a potential therapeutic target.

## INTRODUCTION

Hepatocellular carcinoma (HCC) is the fifth most common cancer in men and the seventh in women worldwide [1]. HCC is now the most common cancer in rural areas of China and the second most common in urban areas [2]. Recent research has supported that human cervical cancer proto-oncogene (HCCR) is a specific and sensitive biomarker independent of serum alpha-fetoprotein (AFP) for the diagnosis of HCC [3–5]. Several studies report that normal liver tissues show minimal, or no, HCCR expression, whereas expression is much higher in individuals with chronic hepatitis, liver cirrhosis (LC), or small and large HCC [5, 6].

*HCCR* gene was first identified as a molecular that is aberrantly and highly expressed in cervical cancer [7]. The HCCR protein contains a domain that is homologous to the mitochondrial leucine zipper-EF-hand-containing transmembrane protein 1 (LETM1) and is localized at the outer membrane of mitochondria [8–10]. Studies in cultured cells and transgenic mice confirmed that HCCR is an oncogene [7, 11]. HCCR inhibits apoptosis [8] and promotes trans-differentiation [12], in part by negatively regulating the tumor suppressor p53 [7] and the HCCR-1 binding protein deleted in polyposis 1 (DP1) [13]. Few studies have examined the upstream regulators of HCCR. Indeed, only two have been identified to date: the PI-3K/Akt pathway [14, 15] and the TCF/β-catenin pathway [16].

Hepatitis B virus (HBV) is the primary causative agent of liver cirrhosis and HCC [2]. HBV infection is very common in regions with high HCC prevalence, and as many as 80–90% of HCC cases occur in HBV-positive individuals [17]. The incidence of HCC is about 100 times higher in HBV carriers than in HBV-negative individuals [18]. The molecular mechanisms underlying the effects of HBV on HCC tumorigenesis have been extensively studied, although the evidence is conflicting. A clear consensus has yet to be reached. The HBV X protein (HBx) plays an important role at all stages of HBV infection by transactivating numerous cellular signaling pathways. However, different experimental methods have led to the identification of many different HBx functions [19],

HCCR is highly expressed in breast, liver, lung, stomach, colon, pancreas, and kidney cancer and in leukemias and lymphomas [7], suggesting that it plays a stem-line role for the initiation of tumor development [20]. According to the fact that the expression of HCCR gradually increases during the development of HCC, we speculated that some unknown factor might stimulate HCCR expression in liver and HCCR expression might correlate with the initiation and development of HCC. Therefore, our current study aims to explore the regulation mechanism(s) and function of HCCR in the development of HCC.

## RESULTS

### Up-regulation of HCCR expression in hepatocytes correlates with HBV replication

**Figure 1:**
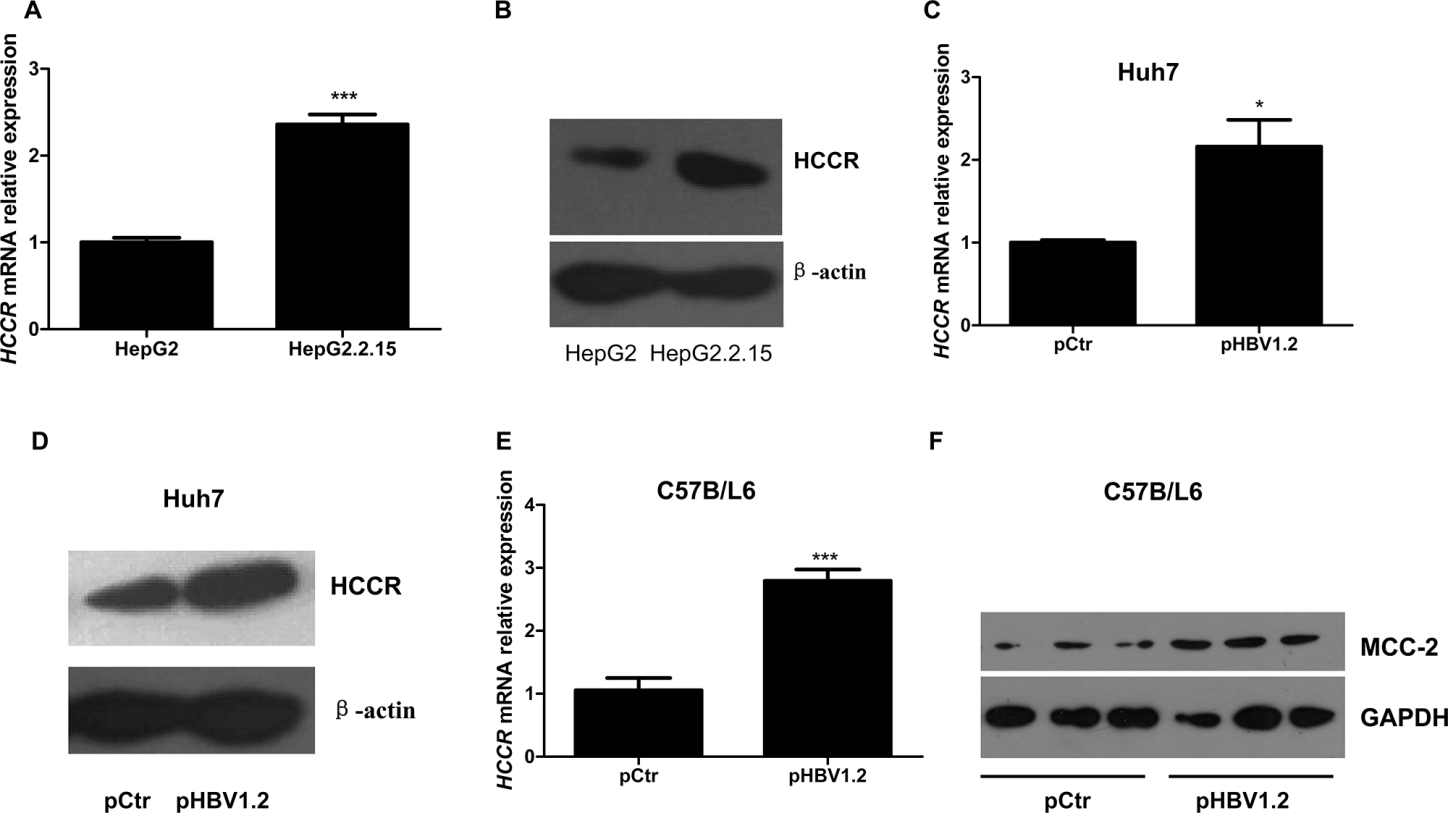
HBV upregulates HCCR expression at the transcriptional level both *in vitro* and *in vivo* Quantitative RT-PCR **A, C, E.** and western blotting **B, D, F.** were performed to examine the relative expression of endogenous HCCR mRNA and protein in HepG2 cells and HepG2.2.15 cells (A, B), in Huh7 cells transiently transfected with pHBV1.2 or an empty vector (pCtr) (C, D). Expression of the mouse HCCR homolog, MCC-2, was also examined in liver tissue from C57BL/6 mice (taken 7 days after mice received a hydrodynamic injection of pHBV1.2 or pCtr (E, F). Each assay was performed in triplicate and the expression level HCCR mRNA was normalized to that of GAPDH. GAPDH or actin was used as a loading control for western blotting. Data are expressed as the mean ± SEM. *P < 0.05 and ***P < 0.001.

HBV is the major causative agent of HCC; therefore, we tested whether HBV influences the expression of HCCR. HBV-expressing hepatocytes, HepG2.2.15, which has been stably transformed with two copies of the HBV genome into human hepatoblastoma HepG2, and its parental cell line HepG2 were used in our research [21]. Real-time RT-PCR and Western blotting were used to detect endogenous HCCR mRNA and protein expression, respectively, in HepG2.2.15 cells and HepG2. As shown in Figures 1A and 1B, both HCCR mRNA and protein levels were markedly higher in HBV-expressing (HepG2.2.15) hepatocytes than in HBV-free hepatocytes (HepG2). Next, to find out whether alterations in HCCR expression are an early or late event following HBV expression, we transiently transfected the human hepatocyte cell line Huh-7 with pHBV1.2, a expression vector contains 1.2 fold genome HBV, or a control vector, and then examined HCCR expression 48 h later. As shown in Figures 1C and 1D, both HCCR mRNA and protein levels were markedly up-regulated in cells transfected with pHBV1.2. We also examined whether HBV promoted the expression of HCCR *in vivo* by examining the expression of a mouse homolog of human HCCR, MCC-32, in mouse liver 7 days after the hydrodynamic injection of the HBV 1.2 expression vector, or a control vector. The results showed that HBV increased the expression of MCC-32 mRNA and protein expression in the liver (Figures 1E and 1F).

### HBx promotes the expression of HCCR at the transcriptional level by inducing HCCR promoter activity

**Figure 2:**
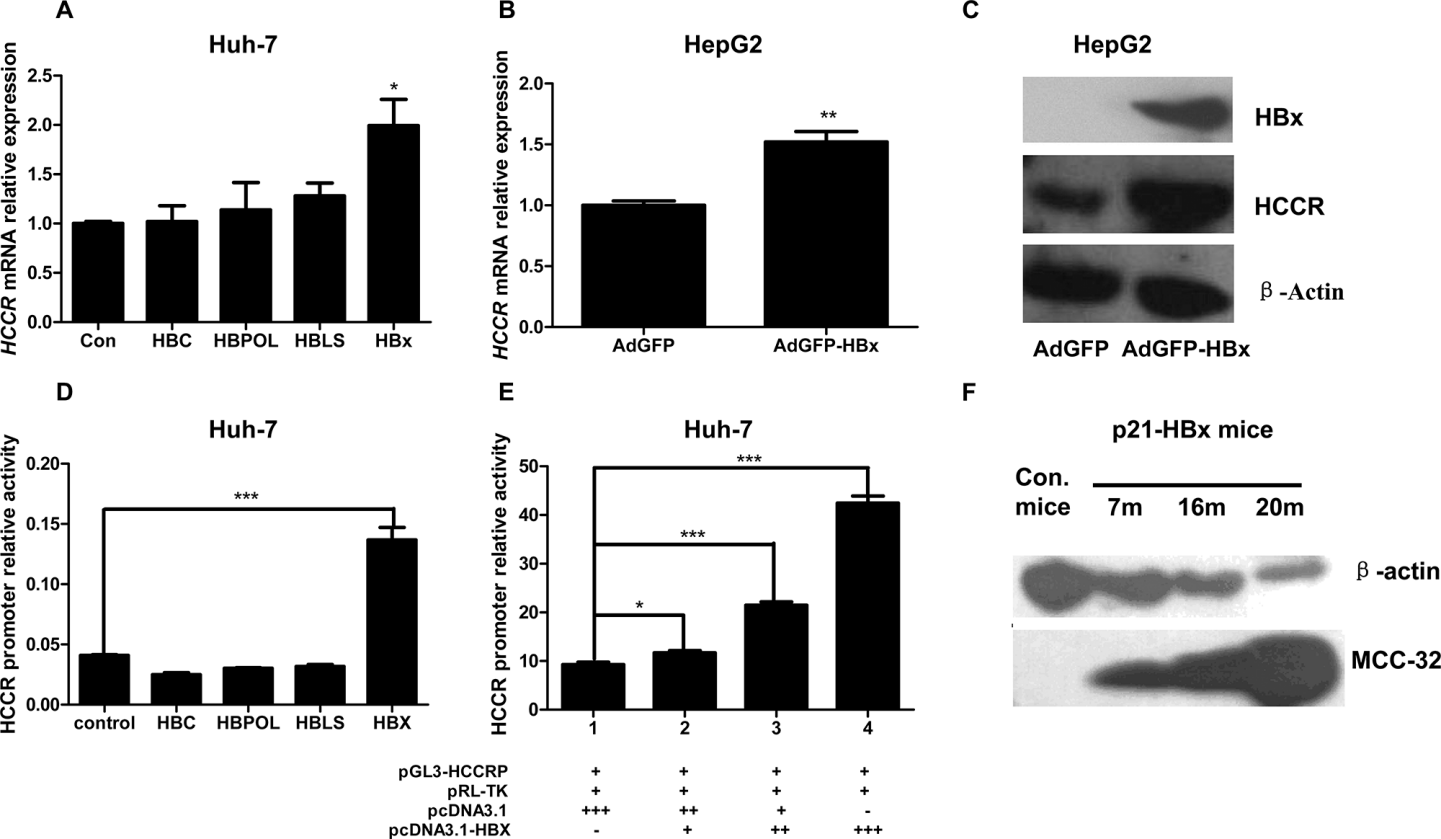
HBx upregulates HCCR expression by enhancing HCCR promoter activity **A.** Huh-7 cells were transiently transfected with one of four different HBV viral factors (HBVLS, HBV core, HBV POL, or HBx) or with an empty vector, and endogenous HCCR expression was measured by real-time RT-PCR 48 h later. **B, C.** HepG2 cells were infected with AdGFP-HBx or AdGFP and levels of endogenous HCCR mRNA were measured by RT-PCR (B) and the levels of HBx, endogenous HCCR, and β-actin proteins were measured by western blotting (C) **D, E.** A luciferase reporter assay was used to measure HCCR promoter activity in Huh-7 cells transfected with luciferase reporter vectors (pGL3-HCCRP and pRL-null) and the indicated expression plasmids. Dual luciferase activity was measured 48 h after transfection. **F.** Liver samples from HBx transgenic and control C57/BL6 mice (aged 7, 16, or 20 months) were examined by western blotting with anti-HCCR (polyclonal) and anti-β-actin antibodies. Each assay was performed in triplicate. The expression HCCR mRNA was normalized to that of GAPDH. Data are expressed as the mean ± SEM. *P < 0.05, **P < 0.01, and ***P < 0.001.

To identify the HBV factor responsible for up-regulating HCCR in hepatocytes, Huh-7 cells were transfected with pcDNA3.1 vectors containing genes encoding the HBV core protein (HBc), large surface protein (HBLS), viral polymerase (HBPol), or X protein (HBx). Overexpression of HBx increased the expression of HCCR mRNA, while the overexpression of other proteins had no effect (Figure 2A). Next, HepG2 cells were infected with a recombinant adenovirus (AdGFP-HBx), and HCCR mRNA level was measured 48 later. The results showed that HCCR mRNA levels were markedly higher in AdGFP-HBx-infected cells than in control (AdGFP)-infected cells (Figure 2B). Similarly, HCCR protein levels were significantly higher in AdGFP-HBx-infected HepG2 cells than AdGFP-infected cells (Figure 2C).

Previous studies have shown that HCCR promoter activity is high in K562 cells, weak in HEK 293 cells, and undetectable in A549 cells [14]. HCCR promoter activity in HCC cells has not been examined. Therefore, a luciferase reporter vector, pGL3-HCCR_P-474-+30_, was constructed and transfected into Huh-7 cells together with the internal control reporter vector pRL-null. We then examined HCCR promoter activity in these cells. To test whether the HBV factor up-regulates of the expression through influencing the activity of HCCR promoter, we used the HCCR promoter reporter system to conduct parallel experiments involving pcDNA3.1-based expression vectors containing HBC, HBPol, HBLS, and HBx. Only HBx increased HCCR promoter activity in Huh-7 cells (Figure 2D). Additional transfection of pcDNA3.1-HBx + pcDNA3.1 resulted in an HBx dose-dependent increase in reporter activity (Figure 2E). When taken together, these results show that the HCCR promoter is active in Huh-7 cells and that its activity is increased by HBx.

A previous study generated an HBx transgenic mouse model to study the role of HBx in HCC tumorigenesis; these mice develop HCC around 20 months of age [22]. To determine whether HBx would up-regulate HCCR *in vivo*, we examined the expression of HCCR homolog MCC-32 in the liver of these HBx transgenic mice at different ages. Liver tissues were taken from HBx transgenic mice and non-transgenic controls aged 7, 16, or 20 months, homogenized, and analyzed by Western blotting. The results showed that MCC-32 was expressed at low levels in non-transgenic mice; however, its expression in HBx transgenic mice increased with age and HCC development (Figure 2F).

### The TCF/β-catenin signaling pathway is involved in the HBx-promotion HCCR expression

**Figure 3:**
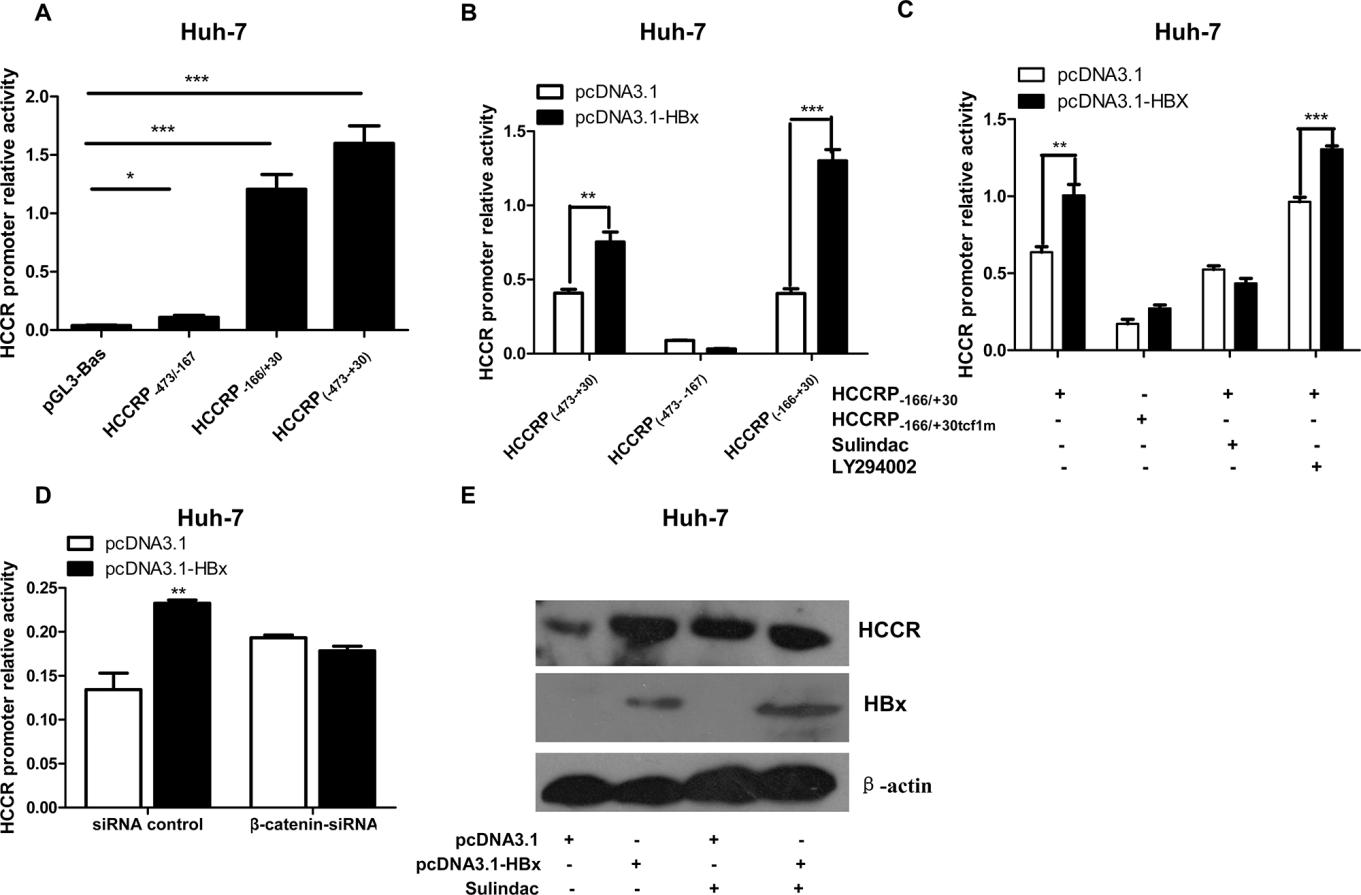
The TCF/β-catenin signaling pathway plays a role in the HBx-mediated regulation of HCCR mRNA expression **A.** Luciferase reporter assay was used to measure the activity of different regions within the HCCR promoter. Reporter vectors containing HCCRP_-474/+30_, HCCRP_-474/−167_, and HCCRP_-166/+30_ were transfected into Huh-7 cells along with the pRL-null vector. **B.** Luciferase reporter assay showing the impact of HBx expression (via pcDNA3.1-HBx) on the activity of the HCCRP_-166/+30_ reporter vectors; **C, D.** The same setup as that in B, but this time the reporter vector HCCRP_-166/+30_ and the tcf-1 site-mutated HCCRP_-166/+30_ (HCCRP_-166/+30tcf1m_) were examined. Huh-7 cells were treated with sulindac (a Wnt/β-catenin pathway inhibitor; final concentration, 600 μM) at 6 h post-plasmid transfection, or with LY294002 (a PI3K/AKT inhibitor; final concentration, 33 μM (C), or with a siRNA targeting β-catenin (D), and then reporter activity was assayed. **E.** Huh-7 cells were infected with AdGFP or AdGFP-HBx and then treated (or not) with 600 mM sulindac for 48 h. The expression of HCCR, HBx, and actin was then examined by western blotting. Each assay was performed in triplicate. Data are expressed as the mean ± SEM. *P < 0.05, **P < 0.01, and ***P < 0.001.

To identify the regions within the HCCR promoter that are critical for its activity, we prepared three luciferase reporter vectors (pGL3-): one harboring the full-length promoter (HCCRP_-474/−167_) and another two harboring two truncated HCCR promoters: HCCRP_-474/−167_ and HCCRP_-166/+30_. The three pGL3-HCCRP vectors were separately transfected into Huh-7 cells, along with pRL-null. A reporter activity assay showed that region − 166 to +30 of the HCCR promoter accounts for the majority of HCCR promoter activity (Figure 3A).

HBx increased the activity of the full promoter (HCCRP_-474/+30_) and HCCRP_-166/+30_, but had no effect on the activity of HCCRP_-473/−167_ (Figure 3B), indicating that HBx targets the − 166 to +30 region of HCCR promoter.

The − 166 to +30 region of the HCCR promoter contains two TCF binding sites (tcf). Tcf-1, which is located in region −26 to +4, is a major element responsible for HCCR-1 expression in K562 cells [16]. Therefore, we constructed a tcf-1 site-mutated reporter vector, pGL3-HCCRP_-166/+30tcf1m_, and transfected it into Huh-7 cells along with pcDNA3.1-HBx and pRL-null. The results of the reporter assay showed that mutating the tcf-1 site essentially abolished the effects of HBx on the HCCRP_-166/+30_ promoter region (Figure 3C). This suggests, in turn, that the TCF/β-catenin signaling pathway is involved in the HBx-induced promotion of HCCR expression in hepatocytes. This was confirmed by the results of the dual luciferase reporter assay, which showed that the HBx-induced promotion of HCCR promoter activity was abrogated by sulindac, an inhibitor of the Wnt/β-catenin signaling pathway (Figure 3C), and by a siRNA oligo targeting β-catenin (Figure 3D). Ly294002 (Figure 3C), a PI3K/Akt pathway inhibitor, had no effect. In addition, sulindac abrogated the HBx-induced increase in HCCR protein expression in Huh-7 cells (Figure 3E).

### Up-regulating HCCR correlates with HBx-induced growth of hepatocytes

**Figure 4:**
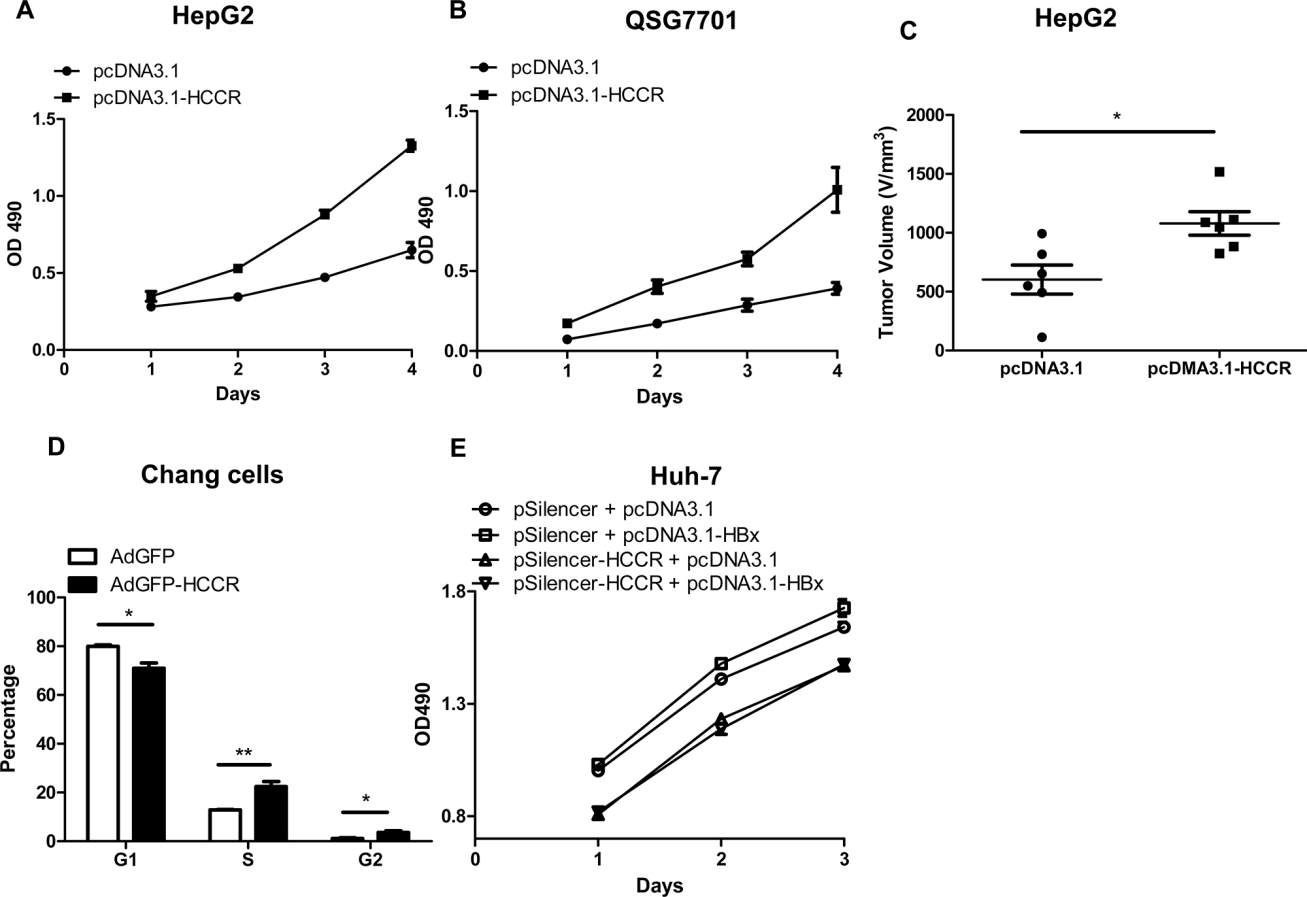
Upregulated HCCR plays a role in HBx-induced hepatocyte growth **A, B.** The effect of HCCR overexpression on the growth of HepG2 cells (A) and normal liver (QSG7701) cells (B) and was assessed in an MTT assay. **C.** The effect of HCCR overexpression on tumor volume of HepG2 xenografts in nude mice. Cells were stably transfected with pcDNA3.1-HCCR or pcDNA3.1. **D.** The effect of HCCR overexpression on the cell cycle status of Chang liver cells was analyzed by flow cytometry at 48 h after infection with AdGFP-HCCR or control virus (AdGFP). **E.** The effects of HCCR suppression on HBx-induced growth of Huh-7 was assessed in an MTT assay. Cells were transiently transfected with pcDNA3.1-HBx or pcDNA3.1 plus pSilencer-HCCR or pSilencer. Assays were performed in at least triplicate. Data are expressed as the mean ± SEM. *P < 0.05, **P < 0.01.

To study the effect of HCCR expression on hepatocytes, we stably transfected HepG2 and QSG7701 cells with pcDNA3.1-HCCR, or pcDNA3.1, resulting in the overexpression of HCCR. HCCR overexpression in pcDNA3.1-HCCR-transfected cells was confirmed by Western blotting (data not shown). Cell proliferation was measured daily by MTT assay. The proliferation of HCCR-overexpressing HepG2 (Figure 4A) and QSG7701 (Figure 4B) cells was significantly higher than that of control cells.

We then invesgated whether HCCR promoted the proliferation of hepatocytes *in vivo*, by injecting HepG2 cells stably transfected with pcDNA3.1-HCCR, or the control pcDNA3.1 into BALB/C nude mice. Tumor dimensions were measured and the volume was calculated 4 weeks later. The results showed that xenograft growth in the group stably transfected with pcDNA3.1-HCCR was significantly faster than that in the control group (Figure 4C).

To assess the impact of HCCR overexpression on hepatocytes cycle, we next infected Chang liver cells with AdGFP-HCCR and AdGFP. Cells were collected at 48 h post-infection, and cell cycle status was examined by flow cytometry after PI staining. The percentage of AdGFP-HCCR cells in S and G2 phase was significantly higher than that of AdGFP (Figure 4D). This is consistent with the cell proliferation results above and suggests that HCCR promotes cell cycle progression.

To elucidate whether HCCR plays a role HBx-mediated carcinogenesis, Huh-7 cells were transfected with the indicated plasmids, and cell proliferation was measured at the indicated times in an MTT assay. The results demonstrate that silencing HCCR expression abolished the HBx-mediated proliferation in Huh-7 cells (Figure 4E). Taken together, these results suggest that HCCR plays a role in the HBx-mediated growth of hepatocytes.

### HCCR enhances the migration and invasion of hepatocyte, and represses its adhesion

**Figure 5:**
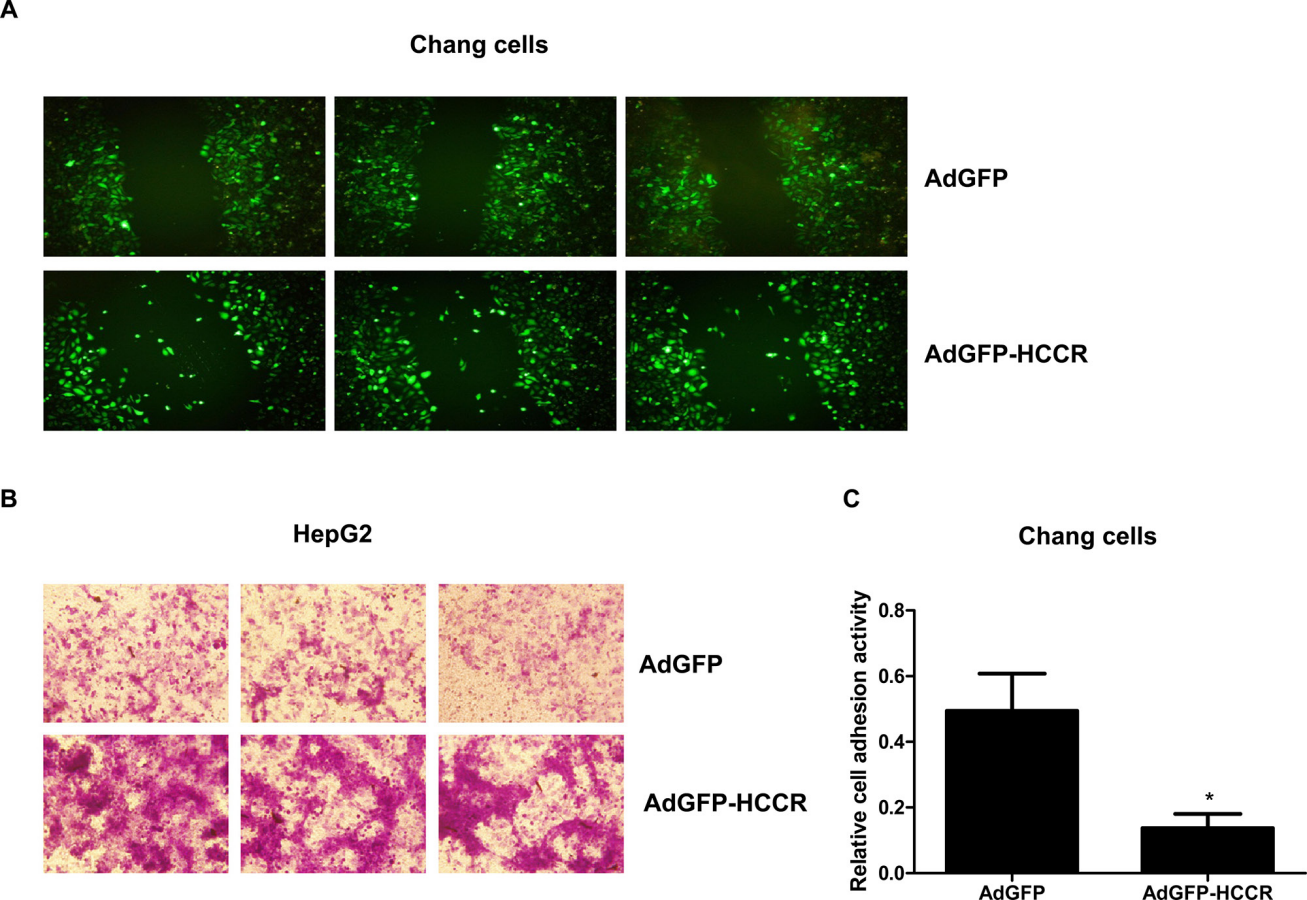
HCCR affects the migration, invasion and adhesion of hepatocytes **A.** Chang liver cells were infected with AdGFP-HCCR or AdGFP. Wounds were crated in a monolayer of cells at 12 h post-infection and fluorescence imaging performed 24 h later. **B.** HepG2 cells were infected with AdGFP or AdGFP-HCCR and then seeded into the upper chambers of 24-well Transwell plates coated with Matrigel. After 24 h, the cells that invaded through the Matrigel layer to the underside of the membrane were stained with crystal violet. **C.** Chang liver cells were infected with AdGFP or AdGFP-HCCR and then seeded into the Matrigel/BSA-coated wells of 96-well plates and incubated at 37°C for 1 h. After washing with PBS (to remove non-adherent cells), the relative cell adherent activity was analyzed as described in methods. Assays were performed in triplicate. Data are expressed as the mean ± SEM. *P < 0.05.

To investigate the biological role(s) of HCCR in HCC, we used an *in vitro* wound-scratch model to examine the migration of AdGFP-HCCR-infected Chang liver cells and compared it with that of control (AdGFP-infected) cells. We found that a significant number of AdGFP-HCCR-infected cells migrated into the “wound”, in contrast to almost none of the AdGFP-infected cells (Figure 5A), suggesting that overexpression of HCCR induces cell migration. Next, we used a Matrigel assay to examine the effects of HCCR on the invasiveness of HepG2 cells. As shown in Figure 5B, the number of AdGFP-HCCR-infected cells migrating through the Matrigel was higher than that of AdGFP-infected cells. To investigate the effects of HCCR on hepatocyte adhesion to the extracellular matrix, HepG2 cells were infected with AdGFP-HCCR, or AdGFP, and examined in an adhesion assay. The results showed that HCCR expression markedly inhibited the adhesion of HepG2 cells (Figure 5C).

Cell cycle progression, migration, and invasion, together with reduced adhesion, are key features of cancer cells; therefore, these results suggest that the up-regulation of HCCR plays an important role in HBV-induced tumorigenesis and metastasis of liver cancer cells.

### HCCR represses the E-cadherin promoter to reduce E-cadherin expression

**Figure 6:**
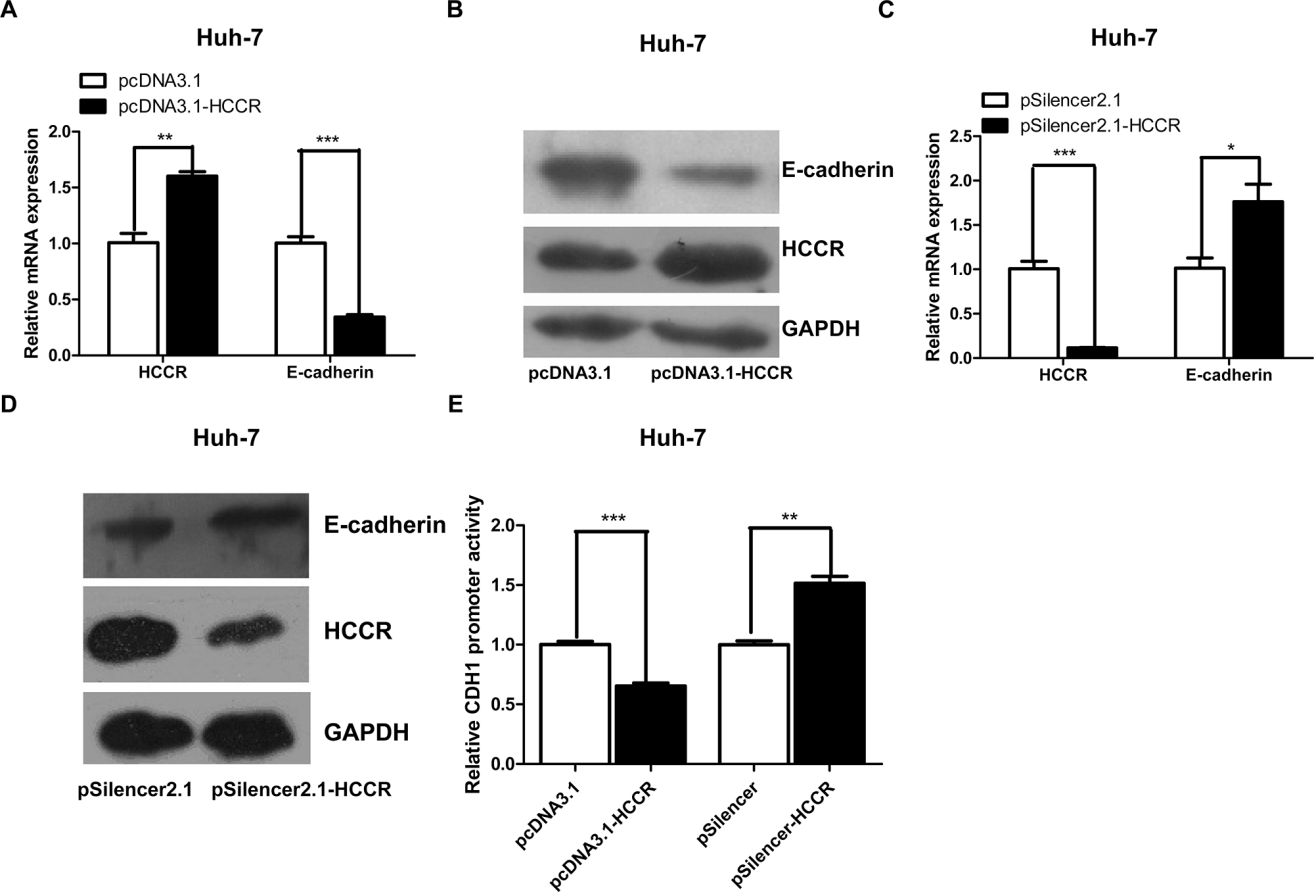
HCCR represses the expression of E-cadherin through its promoter **A, B.** Relative expression of HCCR and E-cadherin mRNA and protein in Huh-7 cells 48 h after transfection with pcDNA3.1-HCCR or a control vector. **C, D.** Relative expression of HCCR and E-cadherin mRNA and protein in Huh-7 cells 48 h after transfection with pSilencer-HCCR or a control vector. **E.** E-cadherin promoter activity in Huh-7 cells transfected with pGL3-E-cadherinP plus pcDNA3.1-HCCR/pcDNA3.1, or with pSilencer-HCCR/pSilencer. Relative expression of HCCR and E-cadherin mRNA was normalized to that of GAPDH. Assays were performed in triplicate. Data are expressed as the mean ± SEM. *P < 0.05, **P < 0.01 m and ***P < 0.001.

A previous study showed that HCCR transgenic mice develop metastatic breast cancer [11]. The results of the present study also show that HCCR promotes cell migration and invasion, and reduces cell adhesion, all hallmarks of cancer. To examine the molecular mechanism(s) underlying the effects of HCCR on hepatocytes, we next compared the mRNA expression profile of HepG2 cells stably transfected with pcDNA3.1-HCCR with that of cells transfected with pDNA3.1 (data not shown). E-cadherin, a key adhesion molecule, which showed decreasing expression in cells overexpressing HCCR, was selected for further analysis. In order to examine the relationship between HCCR expression and E-cadherin expression in liver cells, Huh-7 cells were transfected with pcDNA3.-HCCR. After 48 h, the cells were collected, and the relative expression of HCCR and E-cadherin mRNA (Figure 6A) and protein (Figure 6B) was examined by real-time PCR and Western blotting, respectively. The results showed that overexpressing HCCR suppressed E-cadherin expression at both the mRNA and protein levels. Next, the effect of decreased HCCR expression on E-cadherin expression was more closely examined. Compared with a scrambled siRNA control (pSilencer2.1), an HCCR siRNA vector (pSilencer2.1-HCCR) reduced the endogenous expression of HCCR mRNA (Figure 6C) and protein (Figure 6D) in Huh-7 cells. The results also showed that pSilencer2.1-HCCR induced a significant increase in E-cadherin mRNA (Figure 6C) and protein (Figure 6D). Next, Huh-7 cells were transfected with a reporter plasmid containing the E-cadherin promoter (pGL3-E-cadherinP) together with an overexpression vector (pcDNA3.1-HCCR /pcDNA3.1), or a suppression vector (pSilencer2.1/pSilencer2.1-HCCR), and pRL-null. The results showed that overexpressing HCCR reduced the activity of the E-cadherin promoter, whereas suppressing HCCR expression increased the promoter activity (Figure 6E). Thus, HCCR represses the expression of E-cadherin by suppressing the activity of its promoter.

## DISCUSSION

**Figure 7:**
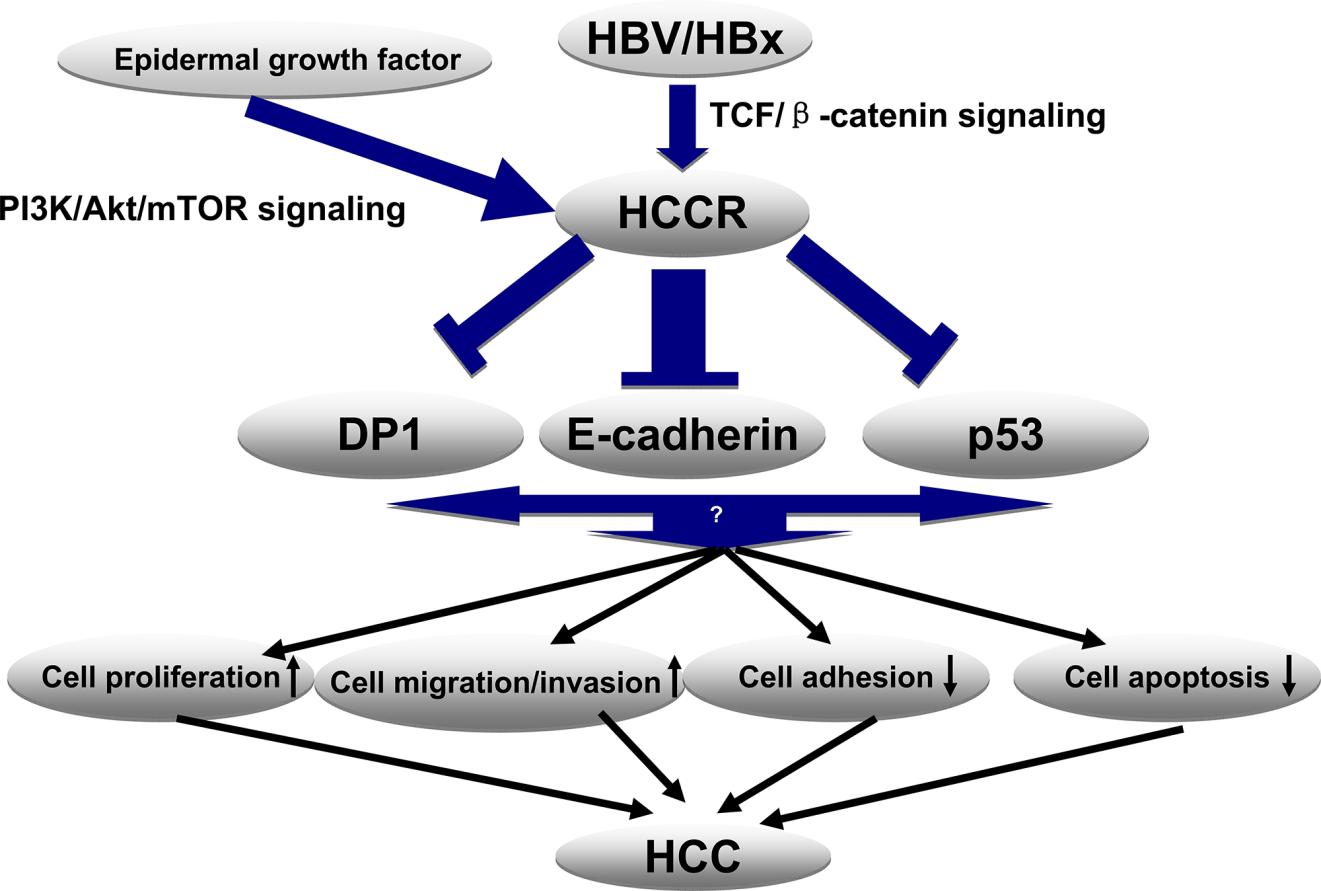
Proposed model for the molecular mechanism underlying HBx- induced HCCR expression and HCC development HBx activates Wnt/β-catenin signaling by promoting β-catenin stabilization and its subsequent nuclear localization. Nuclear-localized TCF and its cofactor (β-catenin) bind the Tcf1 site on the HCCR promoter and activate the expression of HCCR. Next, HCCR suppresses E-cadherin through its promoter (in addition to downstream molecules p53 and DP1). Reduced expression of E-cadherin, p53, and DP1 promotes hepatocyte proliferation, migration, and invasion and represses apoptosis and cell adhesion to the extracellular matrix. These changes contribute to malignancy and to the development of HCC.

The present study shows that HBV, specifically the HBx protein, up-regulates HCCR transcription in human hepatocytes via the TCF /β-catenin pathway by targeting the tcf-1 site within the HCCR promoter region. The PI-3K/Akt pathway plays no role in HBx up-regulation. Furthermore, overexpressing HCCR in cultured hepatocytes, promotes cell proliferation, migration and invasion, while, at the same time, suppressing cell adhesion. Suppressing HCCR expression abolished the effect of HBx-induced hepatocyte growth. Thus, up-regulated HCCR may play an important role in HBV-induced tumorigenesis and metastasis. When we examined the mechanism underlying HCCR-induced carcinogenesis more closely, we found that HCCR represses the expression of E-cadherin, a key cell adhesion molecule, at both the transcription and translational levels by suppressing its promoter. HCCR down- regulation of E-cadherin expression may constitute, at least in part, the mechanism underlying the HCCR-mediated induction of hepatocyte migration, invasion, and metastasis (Figure 7).

This is the first study to establish a causal link between a known cancer- promoting agent (HBV) and a known oncogene (HCCR). In addition, we are the first to report that HCCR expression in hepatocytes is regulated by the TCF/β-catenin pathway. Previous studies showing that HCCR is regulated by this pathway were conducted in non-hepatocyte cell lines (K562 and HEK293) [16]. In addition to p53 and DP-1 (“deleted in polyposis 1”, an HCCR-1-interacting molecule), we have also identified E-cadherin as a molecule that acts downstream of HCCR, providing yet another link between HCCR expression and tumor metastasis.

Previous studies show that HBx plays an essential role in activating the Wnt/β-catenin signaling pathway in hepatoma cells by promoting β-catenin stabilization and its subsequent nuclear localization [23, 24]. TCF/β-catenin also plays an important role in the expression of HCCR in HEK293 and K562 cells [16], which are non-hepatocyte cell lines. Meanwhile, the same group reported that the PI-3K/Akt pathway regulates HCCR expression in NIH/3T3 and K562 cells [14]. Therefore, our finding that HBx promotes the expression of HCCR in hepatocytes via the TCF/β-catenin pathway, rather than the PI-3K pathway, not only provides the first evidence of a direct link between HBx and HCCR, but also provides a set of cell-context-specific regulatory interactions.

Several studies indicate that HCCR expression increases as liver cells gradually progress from LC to HCC [5]; thus, HCCR expression may be a useful marker for HCC progression and/or prognosis [3]. We have made similar observations in clinical samples. An HBx transgenic mouse model was established by introducing the HBx gene into the p21 locus. Around 60% of p21-HBx transgenic mice develop hepatocellular cancer within 18 months after birth, which mimics the development of HCC in humans after chronic HBV infection [22]. Here, we found that the expression of an HCCR homolog, MCC-32, in the livers of HBx transgenic mice followed a similar pattern, i.e., MCC-32 expression increased as the mice aged and developed tumors. We also found that the up-regulation of HCCR mediates HBx-induced growth of hepatocytes. Taken together, these data suggest that HCCR plays an important role in the progression of LC to HCC, thereby confirming prior studies, as noted above.

E-cadherin, which promotes cell-cell contact and suppresses the malignant invasion and metastasis of epithelial cells [25], is associated with the invasiveness of HCC cells [26]. At the molecular level, HCCR promotes cancer by negatively regulating the tumor suppressor p53 and DP-1. Here, we found that HCCR also represses E-cadherin expression by targeting its promoter. Down-regulating E-cadherin expression may promote hepatocyte migration and invasion and HCC metastasis. E-cadherin expression is frequently altered in HCC [27, 28], particularly in HBV-related tumors [28, 29]. The HBx protein represses E-cadherin expression by activating DNA methyltransferase 1 [30, 31]. The results of the present study show that HBx increased HCCR expression and that HCCR, in turn, suppressed E-cadherin expression, suggesting that HCCR may also be involved in the mechanism of HBx regulation E-cadherin. This will be the subject of future studies.

HCCR is localized to the outer membrane of mitochondria and is involved in the formation of an active mitochondria K+/H+ exchanger [32], which is key to maintaining mitochondrial membrane potential and integrity. Therefore, overexpressing HCCR may improve the energy supply to cells, which explains the widespread increase in HCCR expression in various cancers. The implications of increased HCCR expression are two-fold. First, high levels of HCCR may indicate a poor prognosis. Second, HCCR, by acting as supplier of energy to cancer cells, may be an excellent therapeutic target, similar to the targeting of VEGF which disrupts the blood supply to a tumor [33].

One direct clinical implication of the present study is that high HCCR expression may be a highly accurate marker for HBV-positive LC and HCC. This will be investigated using clinical samples in future studies. The hypothesis that HCCR may be a therapeutic target has some support. For example, matrine, which is currently used in the clinic to treat LC [34] and HCC [35, 36], inhibits cell proliferation, promotes cell differentiation and apoptosis, reduces cancer cell invasion and metastasis, and inhibits HCCR/LETMD1 mRNA and protein expression in cultured HCC cells [37].

This paper focuses on HBx-HCCR interactions in relation to HCC progression and stresses the importance of HCCR in HCC; however, it should be noted that HBV-related HCC pathogenesis is a very complicated process involving the interplay of many factors and that HBx transactivates numerous cellular signaling pathways. Indeed, experimental results derived using different approaches or systems have yielded contradictory results [38]. HBx shows either apoptotic or proliferative activity in a context-dependent manner [38]. The previous study show that HBx promotes the growth of hepatoma cells [39] and the results herein show that HCCR is involved in the HBx-induced proliferation of hepatoma cells. Another study also shows that HBx promotes hepatocyte migration and invasion and inhibits the cell adhesion [40, 41]. Here, we showed that HCCR is also associated with these cellular functions. Therefore, we can assume that HCCR is also involved in HBx-induced migration and invasion and the suppression of cell-cell adhesion. Taken together, these results suggest that HBx promotes the progression of HCC by activating the expression HCCR.

HBV is a non-cytopathic virus, and chronic infection lasts for decades; therefore, whatever the role of HBx may be during the early stage of HBV infection, it is probably quite balanced and controlled and does not drive hepatocytes to proliferate in an uncontrolled manner. It is possible, however, that gradual changes in the cellular context, including increased expression of important stem-line oncogenes, such as HCCR, act together with HBV-derived factors to promote progression to HCC.

In summary, this study demonstrates that HBV, specifically the HBx protein, up-regulates HCCR in hepatocytes through TCF/β-catenin signaling pathway. HCCR correlates with the progression of HBx promoting HCC. Furthermore, the present study identified the molecular mechanism, i.e., repressed E-cadherin expression, which may underlie the biological function of HCCR in HCC. These results improve our understanding of the complex mechanisms by which HBV induces HCC and highlight potential clinical applications of HCCR as a marker for HCC progression and prognosis, as well as a potential therapeutic target.

## MATERIALS AND METHODS

### Plasmids, recombinant adenoviruses and siRNA

The expression vector for the 1.2-fold over-length HBV genome (pHBV1.2), the pcDNA3.1-based expression plasmids for the individual HBV genes (Core, LS, Pol, and HBx), and the recombinant adenoviruses expressing HBV (AdGFP-HBV) and HBx (AdGFP-HBx) have been described previously [42]. Expression plasmid pcDNA3.1-HCCR was constructed by inserting the *HCCR* gene (amino acids (aa) 1–360) into pcDNA3.1(+) (Invitrogen). A recombinant adenovirus expressing HCCR, AdGFP-HCCR, was constructed by inserting the *HCCR* gene in the shuttle vector pAdTrack-CMV, recombined with Ad backbone plasmid pAdEasy-1 vector and packaged in QBI293A, a human embryonic kidney cell line. Various fragments of the human HCCR promoter (HCCRP; nucleotides (nt) − 473 to +30, nt − 473 to − 167, and nt − 166 to +30, relative to the transcriptional start site) and of the E-cadherin promoter (E-cadherin P; nt − 420 to +37, relative to the transcriptional start site) [30] were amplified from the genome of HepG2 cells and separately inserted into the pGL3-basic vector (Promega). Site-directed mutagenesis of the T-cell factor (TCF) binding site within HCCRP_-166/+30_ was performed using the Multipoints Mutagenesis Kit (TaKaRa Biotechnology) as previously described [42]. siRNA oligos targeting β-catenin were synthesized by GenePharma Co. (Shanghai, China) [43]. An HCCR silencing vector was constructed by cloning annealed siRNA oligonucleotides targeting *HCCR* mRNA into pSilencer-2.1-U6 hygro siRNA expression vector (Ambion) [44]. All of the associated primers and siRNA sequences are listed in Supplementary Table S1.

### Cells and tissues

HCC cell derived HepG2, Huh-7 and normal liver cell derived QSG7701, Chang liver cells were obtained from the National Infrastructure of Cell Line Resources of China and maintained in Dulbecco's modified Eagle's medium (DMEM) containing 10% fetal calf serum, 2 mM glutamine, 100 units/ml penicillin, and 100 μg/ml streptomycin. HepG2.2.15 cells were maintained in DMEM containing G418 (0.38 μg/ml). All the cells were maintained in a 5% CO_2_ atmosphere at 37°C. Cells were transfected with the designated plasmids or siRNAs using Lipofectamine™ 2000 (Invitrogen). Stable cell lines were established by transfecting the designated cells with the indicated plasmids followed by selection with G418 (400 μg/ml)

### Animals and ethics statement

pHBV1.2-transfected mouse model: 6-week-old male C57BL/6 mice were transfected by injection with 20 μg pHBV1.2 or control vector (pCtrl) diluted in 1 ml Phosphate Buffered Saline (PBS). Mice were sacrificed at day 7 after inoculation, and their livers were snap frozen in liquid nitrogen and stored at −70°C for subsequent analyses. Liver tissues from HBx gene knock-in transgenic mice (C57BL/6) were prepared as described previously [42].

All animal-related procedures were approved by the Institutional Animal Care and Use Committee of Beijing Institute of Microbiology and Epidemiology (Permit No. BIME 2014–18). The animal studies were conducted in strict accordance with the recommendations in the Guide for the Care and Use of Laboratory Animals.

### Real-time quantitative RT-PCR

To examine the relative expression levels of *HCCR* and *E-cadherin* mRNA, total RNA was extracted from the designated cells or liver tissues using the Trizol reagent (Invitrogen), 1 μg RNA was reverse transcribed using M-MLV reverse transcriptase (TaKaRa), and real-time PCR analysis was performed using Real-time PCR Master Mix (Toyobo) and an Eppendorf Mastercycler *ep Realplex* instrument. Data were expressed as expression relative to that of the housekeeping gene *GAPDH*.

### Western blot analysis

Western blotting was performed according to standard protocols. Briefly, proteins were separated in 8% or 12% SDS-PAGE gels and then transferred onto a PVDF membrane (Millipore). The membrane was probed with antibodies against HCCR (an in-house polyclonal antibody recognizing human HCCR and its mouse homolog, MCC-32, Santa cruze), E-cadherin (Cell Signaling Technology), and GAPDH (Cell Signal Technology). Reactive bands were visualized using an HRP-conjugated secondary antibody and a chemiluminescence reagent (Millipore).

### Dual luciferase reporter assays

To test the activity of the HCCR promoter, Huh-7 cells were transfected with reporter plasmids containing different fragments of the human HCCR promoter together with an internal control reporter vector, pRL-null vector (Promega), and one of the pcDNA3.1 expression constructs or siRNA. Six hours after transfection, cells were treated with 33 μM LY-294002 (Sigma-Aldrich; a PI3 kinase inhibitor) or 600 μM Sulindac (Sigma-Aldrich; a Wnt/β-catenin pathway inhibitor). To examine the effect of HCCR on the activity of the E-cadherin promoter, Huh-7 cells were transfected with pGL3-E-cadherinP together with pRL-null and pcDNA3.1-HCCR/ pcDNA3.1, or pSilencer2.1-HCCR/ pSilencer 2.1. 48 h after transfection, cells were harvested and tested using the Dual Luciferase Reporter Assay System (Promega), according to the manufacturer's instructions.

### Cell cycle analysis

Chang liver cells were plated in 6-well plates at a density of 2.5 × 10^5^ cells per well and infected with AdGFP or AdGFP-HCCR 18 h later. After 48 h of incubation, the cells were washed once with PBS, pelleted by centrifugation, and resuspended in PBS at a density of 1 × 10^5^ cells/500 μl. Each 500 μl aliquot was added to 4.5 ml of cold 70% ethanol, and the cells were fixed for 24 h at 4°C. The cells were then pelleted, resuspended in 400 μl propidium iodide (PI) staining solution (0.1% Triton X-100, 20 μg/ml PI, and 200 μg/ml RNase A), and incubated at 37°C for 30 min before analysis in a flow cytometer (*easyCyte 6HT-2L*; Guava Technologies) using the Cell Cycle program, according to the manufacturer's instructions.

### MTT assay

The designated cells in the logarithmic growth phase were diluted in growth medium (5 × 10^4^/ml) and transferred into triplicate wells in 96-well plates (100 μL/well). An MTT (3-(4, 5-Dimethylthiazol-2-yl)-2, 5-Diphenyltetrazolium Bromide) assay was used to measure the number of viable cells every day for 4 days. All measurements were made in triplicate. Briefly, 20 μL of MTT was added to each well and the plates incubated for 4 h. The culture medium was then removed and the formazan crystals dissolved in 150 μL DMSO. Optical density (OD) was read at 490 nm in an ELISA plate reader (Biotek). The mean OD values were calculated and proliferation curves constructed.

### *In vivo* tumor xenograft study

The *in vivo* function of HCCR on hepatocytes was examined by xenograft assay. Four- to five- week-old female BALB/c nude mice (*n* = 12) were randomly divided equally into two groups. Each mouse was subcutaneously injected with 100 μl 5 × 10^6^ HepG2 cells stably transfected with pcDNA3.1 or pcDNA3.1-HCCR. Four weeks later, tumors dimensions were measured and the volume was calculated using the formula: (larger diameter × (smaller diameter)^2^) /2. All the animal experiments were approved by the Animal Experimentation Ethics Committee.

### Cell migration, invasion, and adhesion assay

Cell migration was examined in a wound-scratch assay. Briefly, Chang liver cells were plated in 6-well plates (2 × 10^5^/well) and grown to 80% confluency. Cells were infected with AdGFP or AdGFP-HCCR for 24 h. The cells in each well were then scraped with a cell scraper to clear a single strip of cells (wound formation). The cells were then rinsed, covered with fresh growth medium and cultured for an additional 24 h. The cells were then washed with PBS, and regrowth was observed under a fluorescence microscope

Matrigel invasion assays were performed in 24-well Transwell plates (Corning) coated with Matrigel (1 mg/ml; BD Biosciences). Briefly, AdGFP- or AdGFP-HCCR-infected HepG2 cells (1 × 10^5^) were seeded into the upper chamber in serum-free DMEM. The lower chamber was filled with NIH3T3-conditioned medium containing 10% FBS. After 24 h, the non-migrating cells in the upper chambers were removed using a cotton swab. Cells that had migrated through the Matrigel layer to the underside of the membrane were stained with crystal violet.

Cell adhesion assays were performed in 96-well plates precoated with Matrigel (50 mg/l stock diluted 1:8 in PBS; 50 μl/well) or 1% bovine serum albumin (BSA) at 4°C overnight. After removing the coating solution, the plates were blocked with blocking buffer (1% BSA in DMEM) for 1 h at 37°C. Chang liver cells were infected with AdGFP-HCCR or control AdGFP for 48 h. The cells were then collected and seeded (1 × 10^5^ cells/well) into triplicate Matrigel/BSA-coated wells and incubated for 1 h at 37°C. After washing with PBS to remove non-adherent cells, cell culture medium was replaced with 120 μl of DMEM/10% FBS containing 20 μl MTT. After 4 h incubation, viable cells were counted as described above. The relative adherence of Chang cells was calculated as follows: (OD of Matrigel-coated wells- OD of bovine serum albumin-coated wells) / (OD of bovine serum albumin-coated wells).

### Statistical analysis

Each experiment was repeated at least three times. Quantitative values were expressed as the mean ± SEM. Between-group comparisons of relative luciferase activity, mRNA levels, and protein levels were made using Student's *t*-test. All analyses were performed using GraphPad Prism 5 software (GraphPad Software, Inc.). A *p*-value < 0.05 was considered statistically significant.

## SUPPLEMENTARY TABLE


