# Supplementary material for: Up-regulation of human cervical cancer proto-oncogene contributes to hepatitis B virus-induced malignant transformation of hepatocyte by down-regulating E-cadherin
Source: Oncotarget. 2015 Sep 4;6(30):29196–208. doi: 10.18632/oncotarget.5039 (PMC4745720; doi:10.18632/oncotarget.5039)
Supplement: Supplementary file 1 [file oncotarget-06-29196-s001.pdf]

SUPPLEMENTARY TABLE

Supplementary Table S1: Details of primers and siRNA used in this study

Primer name	Sequence	Experiments
HCCRP1	GCCACC ATGGCGCTCTCCAGGGTGTGCTG	HCCR cDNA
HCCRP2	TCAGCGCCTTGTCCCAAGGTAGTTG	
HCCRPP1-F(-473—451)	GGTACCGGCTTCACAGCGAGAGAAGCGG	HCCR Promoter
HCCRPP1-R(-167—186)	CTCGAGCTTCACAGCGAGAGAAGCGGGAG	
HCCRPP2-F(-166—146)	GGTACCCCTTCCGCACTTAGTCTTTGAG	
HCCRPP2-R(+10-+30)	CTCGAGCTTCACAGCGAGAGAAGCGGGAG	
HCCRPm1	ACAGCAAAAGCCCGAGCGACCTTCGGT ACC CCAGACTGACTGGAAAGTG	Mutation in TCF1 binding site of HCCR promoter
HCCRPm2	CCCGCTTCTCTCGCTGTGAAGCTCGAGC GGC TAAGGCACGCGCCACTTT	
HCCRPm3	CCGCACTTAGTCTGGTAGTCCTTCCCTCTC	
Hu-HCCRrealp1	TCGTTTCTTGGGTCGTCATTT	Real time PCR for HCCR of human
Hu-HCCRrealp2	TATTCTTCTAGCCTTTTTGGCATCA	
Hu-GAPDH realp1	TGGGTGTGAACCATGAGAAGTATG	Real time PCR for GAPDH of human
Hu-GAPDH realp2	ACTGTGGTCATGAGTCCTTCCA	
Mus-MCC32 realp1	CCGCTTCTATGCCTTATACACAAC	Real time PCR for MCC32 of mouse
Mus-MCC32 realp1	TTGTTTCCACATGTCTGCCTTT	
Mus-GAPDH realp1	TGCACCACCAACTGCTTAGC	Real time PCR for GAPDH of mouse
Mus-GAPDH realp2	GTCTTCTGGGTGGCAGTGATG	
β-catenin-siRNA-sense strand	GGGUUCAGAUGAUAUAAAUTT	β-catenin-siRNA
β-catenin-siRNA- antisense strand	AUUUAUAUCAUCUGAACCCAG	
Hu-HCCRI P1	GATCCAGAAGCTGAGCTGTTTCTCTTCA AGACATATATACAGCTGAGATTCATGT TATGGAAA	HCCR siRNA expression vector
Hu-HCCRI p2	AGCTTTTCCATAACATGAATCTCAGCTG TATATATGTCTTGAAGAGAAACAGCTC AGCTTCTG	
E-cadherinPP1:	GGTACCACCGCTCGAGCCCAGGAGTT	E-cadherin promoter
E-cadherinPP2:	CTCGAGCCGCAAGCTCACAGGTGC	
E-cadherin realp1	TGAAGGTGACAGAGCCTCTGGAT	Real time PCR for E-cadherin of human
E-cadherin realp1	TGGGTGAATTCGGGCTTGTT	
